# Let’s Not Joke about It Too Much! Exposure to COVID-19 Messaging, Attitudes and Protective Behavioral Intentions

**DOI:** 10.3390/healthcare9020122

**Published:** 2021-01-25

**Authors:** Petru L. Curșeu, Andra D. Coman, Oana C. Fodor, Lucia Rațiu, Anton Panchenko

**Affiliations:** 1 Department of Psychology, Babeș-Bolyai University, 400084 Cluj-Napoca, Romania; andracoman@psychology.ro (A.D.C.); oanafodor@psychology.ro (O.C.F.); luciaratiu@psychology.ro (L.R.); antonpanchenko@psychology.ro (A.P.); 2 Department of Organization, Open Universiteit, 6419 AT Heerlen, The Netherlands

**Keywords:** public communication, COVID-19, theory of planned behavior, humor, emotions, attitudes

## Abstract

Our study evaluates the role of exposure to COVID-19 messaging in negativity towards COVID-19 and the intentions to engage in protective behaviors. Building on the theory of planned behavior (TPB), we derive a mediation model and test it in a sample of 737 participants (556 Romanians and 181 Kazakhs). The exposure to general information concerning COVID-19 positively predicts negative attitudes, negative emotions and the emergence of subjective norms in relation to COVID-19, as well as the likelihood of engaging in protective behavioral intentions. The exposure to humoristic communication, however, diluted the positive association between exposure to general information and negative attitudes, as well as negative emotions. The results support the overall predictions of the TPB and report positive associations between negative attitudes towards COVID-19, subjective norms and behavioral control on the one hand, and protective behavioral intentions on the other. Negative emotions significantly predict the likelihood of engaging in protective behaviors. Our results also show that older respondents tend to develop more negative attitudes towards COVID-19, yet they do not report stronger intentions to engage in protective behaviors than younger respondents. An important emergent result shows that time lapse seems to increase negativity towards COVID-19, yet it does not directly increase the likelihood of engaging in protective behaviors. Implications for public health communication related to COVID-19 are discussed.

## 1. Introduction

As the COVID-19 pandemic continues to cause a significant number of deaths, governments across the world struggle to find ways to contain the rapid and wide spread of the virus [1,2]. Public messages are a key persuasive tool used in the fight with the virus [3,4]. Social media also plays an important role in the distribution of these messages [5,6,7,8]. Messages related to COVID-19 vary from messages that provide general information about the pandemic to messages that use humor in relation to the virus and the pandemic. Our study sets out to investigate the interplay of general informative messages with humoristic messages in COVID-19-related communication. In particular, we build on the insights from the theory of planned behavior (TPB) [9], one of the most influential theories used to predict health-related behaviors [10,11,12] to argue that exposure to general information related to COVID-19 generates negative attitudes towards COVID-19 and reveals subjective norms related to the dangers of the pandemic, which in turn generate protective behavioral intentions. Further, we intend to explore the moderating role of behavioral control in the relationship between negative subjective norms and attitudes towards COVID-19 on the one hand and protective behavioral intentions on the other. We extend the TPB in two ways. First, we take into account negative emotions experienced in relation to COVID-19 as an additional plausible mediator in the relationship between exposure to COVID-19 information and protective behavioral intentions. Second, we explore the interaction between exposure to general and humoristic information in the emergence of negative attitudes and subjective norms in relation to COVID-19.

### Theory and Hypotheses

The theory of planned behavior (TPB) [9] is one of the most influential theories used to predict and explain behaviors in a variety of situations [10,12] and in particular to predict protective behavioral intentions in the context of the COVID-19 pandemic [13,14,15,16]. With respect to health and health-related behaviors, the theory has been used extensively and meta-analytical evidence suggests it explains around 66% of the variance in behavioral intentions and about 33% of the variance in health-related behaviors [11]. The three key predictors in the TPB are attitudes (evaluative cognitions related to a target or behavior), subjective norms (perceived social consensus referring to a target or behavior) and behavioral control (perceived extent to which a particular behavior is under one’s control) [9,17]. According to the initial theorizing, the three components of the TPB are conceptually independent [9] and their relevance as behavioral antecedents depends on a variety of factors, ranging from personal autonomy and agency with respect to the targeted behaviors [18] to anticipated affect and moral norms [19]. As most health-related behaviors are subjected to motivational control [11,18] and they are associated with anticipated negative consequence for health and survival, the TPB is particularly relevant as an explanatory framework. In the case of the COVID-19 pandemic, the general message conveyed in public communication across the world presents preventive measures, ranging from washing hands to social distancing [20]. In line with previous research [13,14,15,16], we argue that the intention to engage in these protective behaviors is explained by the attitudes people develop concerning the virus (the set of negative evaluative cognitions concerning the virus and its consequences), the subjective norms they perceive around them in relation to the virus (how do others tend to perceive the danger of the virus, the pandemic and its consequences) and the behavioral control (the ease of performing actions in order to prevent infection with the virus).

The TPB was adapted in recent years to include the role of experienced, as well as anticipated, emotions as one additional predictor of behavioral intentions, next to attitudes and subjective norms [19]. In particular, studies focused on anticipated regret stemming from inaction and meta-analytic evidence shows that regret accounts for an additional 5 to 7 percent of variance in behavioral intentions [18,21]. We further contribute to the TPB by taking into account experienced rather than anticipated emotions in relation to the target. We build on three models that explicitly address the link between emotions and behavioral intentions, namely, on the affect as information theory [22], on the “feeling is for doing” model [23] and on the appraisal tendency framework [24], to ground our hypothesis concerning the link between emotions experienced in relation to COVID-19 and protective behavioral intentions. Based on the affect as information theory [22] and the appraisal tendency framework [24], we argue that the negative emotions experienced in relation to COVID-19 signal danger, and as a consequence are likely to enforce protective behavioral intentions. The feeling is for doing model [23] emphasizes the motivational function of emotions, their impact on goal-directed behavior and, in the context of the COVID-19 pandemic, the negative emotions associated with the viral threat are motivational forces and generate protective intentions. In line with these arguments, we use negative emotions, as well as the three predictors derived from the TPB, to explain the behavioral intentions to protect against the negative consequences of being infected with COVID-19, and we hypothesize the following:

**Hypothesis** **1** **(H1).** *Negative attitudes (H1a), subjective norms (H1b), behavioral control (H1c) and negative emotions (H1d) have a positive association with behavioral intentions to prevent infection with COVID-19.*

The TPB emphasizes the role of belief systems as antecedents of attitudes, subjective norms and behavioral control. Such belief systems emerge from personal experiences and salient information structures that result from exposure to and processing of behavior-relevant information [9]. In the context of the COVID-19 pandemic, information about the virus and pandemics is widely available from public (official) communication channels and social media. In line with the TPB, we argue that exposure to information leads to the development of attitudes towards COVID-19 and it conveys information on the subjective norms. Information disseminated via the official channels emphasizes the fast spread of the virus, its virulence and negative health consequences, especially for the elderly [25]. In a recent study that extended the TPB to explore the antecedents of protective behavioral intentions in the context of the COVID-19 pandemic [16], it was shown that one’s understanding of COVID-19 predicts attitudes and subjective norms in relation to protective behaviors. We argue that exposure to general information about COVID-19 generates negative evaluative tendencies and emotions. Exposure to general information about the COVID-19 pandemic is likely to generate a more accurate representation about the dangers associated with the infection and as such lead to more negative evaluative cognitions (attitudes) and emotions in relation to COVID-19. The widespread dissemination of such information via formal and informal (social media) channels will also establish a sense of what the social norms are with respect to dangers associated with the COVID-19 pandemic. We therefore hypothesize that:

**Hypothesis** **2** **(H2).** *Exposure to general information concerning COVID-19 is positively associated with negative attitudes (H2a), subjective norms (H2b) and negative emotions (H2c) related to COVID-19.*

As we mentioned earlier, information concerning the COVID-19 pandemic is also disseminated via different channels, including various social media channels [6]. A substantial number of the messages, especially the ones distributed via social media, use humor to depict COVID-19 and the pandemic in general. Examples, such as: “Pro-tip: you should wash your hands even when there isn’t a global virus panic”, or “They said a mask and gloves are enough to go to the grocery store. They lied! Everybody else had clothes on”, or “Your grandparents were called to arms. You’re being called to sit on a couch. You can do this!”, abound in social media. Humor is a protective mechanism in stressful situations [26] and most certainly humor is also used as a coping mechanism in the context of the COVID-19 pandemic. Mechanisms used to explain the health benefits of using humor in communication include: the beneficial effects of its biochemical correlates on the immune and cardiovascular systems, its potential to distract attention from traumatic stimuli and reappraise the stressors and its potential to facilitate social connectivity [27,28]. In line with the media polychronicity findings [29], we argue that simultaneous exposure to general information about COVID-19 and humoristic communication about it reduces the depth of information processing for the main informative messages.

We argue that the use of humor in COVID-19-related communication induces a general positive emotional atmosphere that can impact on the development of attitudes via several mechanisms. First, in line with the affect as information theory [22], when making judgments, people use the emotions they experience in relation to a target as cues for their evaluations of the target. Experiencing positive emotions in relation to COVID-19 will lead to more positive attitudes because such positive emotions derived, for example, from joking about COVID-19, are used as evaluative cues. Second, the superficial information processing associated with positive affect [30,31] may reduce the depth of information processing for other COVID-19 (informative) messages. Third, in line with the affective evaluative nature of attitudes [17,32], the positive affective context generated through the use of humor may reduce the negativity (attitudes, subjective norms and emotions) in relation to COVID-19. This argument is also in line with the hedonic contingency hypothesis [33], stating that when experiencing positive moods, people are motivated to maintain them and positive reinforcing contingencies are activated, leading to a tendency to search for hedonic behavioral contingencies. In the context of COVID-19, jokes that may create a positive mood generate a more positive evaluation of the pandemic.

Information may directly trigger the intention to behave in a particular way. For example, communication concerning an imminent danger may signal a social norm and lead to defensive behaviors even in the absence of specific attitudes toward that particular situation. In the context of COVID-19 public communication and wide exposure to information about the virus, we expect that the effect of information on behavioral intentions is (partially) mediated by negative attitudes, negative emotions and subjective norms. In line with these arguments, we state the following hypotheses:

**Hypothesis** **3** **(H3).** *Exposure to humoristic information about COVID-19 is negatively associated with negative attitudes (H3a), subjective norms (H3b) and negative emotions (H3c) related to COVID-19.*

**Hypothesis** **4** **(H4).** *Exposure to humoristic information about COVID-19 attenuates the positive effect of exposure to general information on negative attitudes (H4a), subjective norms (H4b) and negative emotions (H4c) in relation to COVID-19.*

**Hypothesis** **5a** **(H5a).** *Negative attitudes towards COVID-19 mediate the effect of exposure to general information on behavioral intentions to prevent infection with COVID-19.*

**Hypothesis** **5b** **(H5b).** *Subjective norms towards COVID-19 mediate the effect of exposure to general information on behavioral intentions to prevent infection with COVID-19.*

**Hypothesis** **5c** **(H5c).** *Negative emotions experienced in relation to COVID-19 mediate the effect of exposure to general information on behavioral intentions to prevent infection with COVID-19.*

The relationship between the three main predictors of the TPB is complex [17] and their interaction shapes behavioral intentions. As opposed to attitudes and subjective norms that stem from interpersonal communication and one’s belief system, behavioral control was linked to control beliefs that are tied to individual differences (extraversion, conscientiousness [34]). Behavioral control describes the general set of beliefs that a particular behavior can be exercised without difficulty and, as such, can be related to one’s self-efficacy beliefs [17]. In the context of COVID-19, we argue that such behavioral control beliefs moderate the association between attitudes and subjective norms on the one hand and the protective behavioral intentions on the other hand. We expect that if individuals have strong behavioral control beliefs concerning infection with COVID-19, the positive association between their negative attitude and protective behavioral intentions will be stronger. In other words, they perceive themselves as being more capable and willing of acting in line with the negative attitudes towards COVID-19. A similar argument applies to the negative association between the subjective norm and the protective behavioral intention. In line with these arguments, we hypothesize the following:

**Hypothesis** **6a** **(H6a).** *Behavioral control accentuates the positive association between negative attitudes towards COVID-19 and protective behavioral intentions.*

**Hypothesis** **6b** **(H6b).** *Behavioral control accentuates the positive association between the subjective norms and protective behavioral intentions.*

**Hypothesis** **6c** **(H6c).** *Behavioral control accentuates the positive association between negative emotions experienced in relation to COVID-19 and protective behavioral intentions.*

## 2. Methods

### 2.1. Sample and Procedure

We used a cross-national sample composed of 556 Romanian-speaking participants (432 women) from Romania with an average age of about 35 years old and 181 Russian-speaking participants (114 women) from Kazakhstan with an average age of about 34 years old.

### 2.2. Data Collection

Our study is survey based and data were collected using self-reported answers to an online survey. Most of the survey items referred to the TPB dimensions and were developed in line with the suggestions presented in Ajzen [35]. Survey items were translated into Romanian and Russian and we used convenience sampling and a snowball approach to invite participants using social media sites (Facebook, VKontakte) to take part in the study. Participants from different regions of the two countries could take part in the survey, yet we cannot claim that our samples are representative of the two countries. Participation was anonymous and participants could withdraw from the study at any moment. The two countries took similar public health measures in relation the COVID-19 pandemic, Romania declaring a state of national emergency on 14 March 2020 and Kazakhstan taking a similar set of measures on 15 March 2020. Data collection started right after these public health measures were taken in both countries. The variables included in the present study were selected from a more consistent COVID-19 survey and the data were sliced following the recommendations presented in Kirkman and Chen [36]. With the exception of demographic and control variables, there was no further overlap among the variables included in the current study and the ones used in another study based on the same survey.

### 2.3. Measures

*Exposure to general information about COVID-19* evaluated the extent to which participants were exposed to general COVID-19-related information communicated via official websites and it was rated using a single item with a visual aid. We selected a picture (screenshot) from an official governmental website offering general information about the virus and asked the participants: “Please think of the last 14 days and the messages concerning COVID-19 that you were exposed to during this interval. Please estimate the extent to which you were exposed to general informative messages related to CORONAVIRUS as the one presented in the example below”. The example depicted a screenshot from an official media site, presenting general information about COVID-19, as well as instructing readers to use only official sites to gather information related to COVID-19. Answers were recorded on a seven-point Likert scale (1 = never to 7 = several times every day).

*Exposure to humoristic information about COVID-19* evaluated the extent to which participants were exposed to messages using humor in relation to the COVID-19 pandemic and was rated using a single item that was also supported by an image with an example of a COVID-19-related joke. The participants were asked the following: “Please think of the last 14 days and the messages concerning COVID-19 that you were exposed to during this interval. Please estimate the extent to which you were exposed to humoristic messages related to CORONAVIRUS as the one presented in the example below”. The example depicted a humoristic message distributed via social media channels. Answers were recorded on the same scale as the answers for the exposure to general information concerning COVID-19.

*Negative attitudes concerning COVID-19* refer to one’s evaluative cognitions in relation to COVID-19 and were evaluated with a newly developed scale containing 3 items. Because the negative attitudes, behavioral control, subjective norms and behavioral intentions were newly developed scales, we used the instructions from Ajzen [35] on how to create instruments aligned with the prescriptions of the theory of planned behavior. The items are: “Coronavirus (COVID-19) is dangerous”, “It is easy to get infected with Coronavirus (COVID-19)” and “Coronavirus (COVID-19) could kill me”. Answers were recorded on a five-point Likert scale (1 = completely disagree to 5 = fully agree) and Cronbach’s alpha for this scale was 0.73. As this is a newly developed and rather short scale, we relied on Hayes and Coutts [37] to compute the omega (a reliability index derived from confirmatory factor analysis) for this scale and the value was 0.73, showing sufficient internal consistency of the items.

*Subjective norms related to COVID-19* refer to one’s beliefs that relevant others hold negative views in relation to COVID-19 and were evaluated with three items, “People close to me consider that the new coronavirus (COVID-19) is dangerous”, “People close to me consider it easy to get infected with the new coronavirus (COVID-19)” and “People close to me consider that the new coronavirus (COVID-19) can kill”. Answers were recorded on a five-point Likert scales with the same anchors as for the negative attitudes. Cronbach’s alpha for this scale was 0.82 and omega was also 0.82, showing good internal reliability of the scale.

*Emotions in relation to COVID-19* were evaluated using a gradient scale presenting emotions organized on a continuum ranging from positive to negative emotions: “How would you best describe your emotions in relation to COVID-19?”. Answers were recorded on a 7-point Likert scale with the following values: 7 = delighted, 6 = pleased, 5 = mostly satisfied, 4 = mixed, 3 = mostly dissatisfied, 2 = unhappy, 1 = terrible. As such, the item evaluates ordered emotions based on their valence on a continuum ranging from positive emotions (delighted) to negative emotions (terrible) related to COVID-19. We recoded the scores in such a way that a high score indicates negative emotions in relation to COVID-19.

*Behavioral control* refers to the perceived difficulty of engaging in protective behaviors against COVID-19 and it was evaluated with three newly developed items: “If I set my mind to it, I can protect myself easily against the new coronavirus (COVID-19)”, “It is up to me to avoid contact with persons likely to be infected with the new coronavirus (COVID-19)” and “I can easily avoid public spaces that are possibly contaminated with the new coronavirus (COVID-19)”. Cronbach’s alpha for this scale was 0.78 and omega was 0.78, indicating sufficient internal reliability of this scale.

*Protective behavioral intentions against COVID-19* were also evaluated with a newly developed scale with four items related to the protective measures indicated in public communications across the globe. In line with the protective behavior dimensions described by Bish and Michie [38], we formulated items for preventive and avoidant behaviors: “I tend to wash my hands more frequently than I used to do before in order to protect myself from the new coronavirus (COVID-19)”, “I intend to avoid contact with persons that seem to have a flu in order to protect myself from the new coronavirus (COVID-19)”, “I tend to avoid public spaces in order to protect myself from the new coronavirus (COVID-19)” and “I am willing to wear a mask in public spaces, in order to protect myself from the new coronavirus (COVID-19)”. Answers were recorded on a five-point Likert scale (1 = completely disagree to 5 = fully agree). Cronbach’s alpha for this scale was 0.81 and omega was also 0.81, indicating good internal consistency of the scale.

### 2.4. Control Variables

As control variables, we collected data on gender (coded as a dummy variable with 0 = men, 1 = women), education (1 = vocational school, 2 = intermediate secondary education/high school, 3 = higher secondary education, 4 = university education (bachelor’s), 5 = university education (master’s), 6 = postgraduate education, 7 = Ph.D.), country (coded as a dummy variable with Romania = 1 and Kazakhstan = 0) and age.

## 3. Results

Means, standard deviations and correlations are presented in Table 1. The moderation hypotheses were tested using ordinary least squares (OLS) regression analyses. All variables that defined products for interaction effects were grand mean-centered before the analyses. In the first regression analysis, negative attitude towards COVID-19 was entered as a criterion and exposure to general information and exposure to humoristic communication as predictors. We also used age, gender, education, country and questionnaire completion day as control variables because these variables are likely to be related to both predictors, as well as the negative attitudes towards COVID-19 and the subjective norms. The results of the regression analysis with the average item scores for the scales are presented in Table 2. The table presents the unstandardized beta coefficients, while further on in the text we will report the standardized beta coefficients from the most complex model (Model 2).

From the control variables, education had a significant positive association (β = 0.08, *p* = 0.03) with the negative attitudes towards COVID-19, with subjective norms (β = 0.09, *p* = 0.02) as well as with the protective behavioral intentions (β = 0.09, *p* = 0.009). Moreover, age has a significant positive association (β = 0.08, *p* = 0.03) with negative attitudes, as well as negative emotions (β = 0.10, *p* = 0.009), in relation to COVID-19, while gender also has a significant positive association with protective behavioral intentions (β = 0.09, *p* = 0.004), such that women report higher protective behaviors than men do. Country of origin also has a significant positive association with negative attitudes (β = 0.37, *p* < 0.001), subjective norms (β = 0.25, *p* < 0.001) and negative emotions (β = 0.35, *p* < 0.001), as well as a significant negative association with behavioral intentions (β = −0.10, *p* = 0.02), such that Romanian-speaking participants reported more negative attitudes, emotions and subjective norms, yet they seemed to report lower scores for protective behavioral intentions in relation to COVID-19 than Kazakh participants. Finally, the survey completion day had a significant positive effect on negative attitudes (β = 0.24, *p* < 0.001) and negative emotions (β = 0.23, *p* < 0.001), as well as on subjective norms (β = 0.19, *p* < 0.001), showing that people who filled out the survey later in the study tended to report more negativity in relation to COVID-19 than people that filled out the survey at the beginning of the study. However, survey completion day did not have a significant association with the intention to engage in protective behaviors (β = 0.04, *p* = 0.38). Therefore, we can conclude that, although people tended to report increasingly negative attitudes, emotions and subjective norms in relation to COVID-19, they did not report more protective behavioral intentions as time passed.

In Hypothesis 1, we expected a positive association between negative attitudes, negative emotions, subjective norms and behavioral control on the one hand and protective behavioral intentions on the other. Significant predictors for protective behavioral intentions are negative attitudes (β = 0.38, *p* < 0.001), negative emotions (β = 0.16, *p* < 0.001) and behavioral control (β = 0.29, *p* < 0.001), all with positive and significant coefficients, supporting Hypotheses 1a, 1c and 1d. Subjective norms predict only marginally protective behavioral intentions (β = 0.06, *p* = 0.07), therefore, Hypothesis 1b received only limited support.

**Figure 1:**
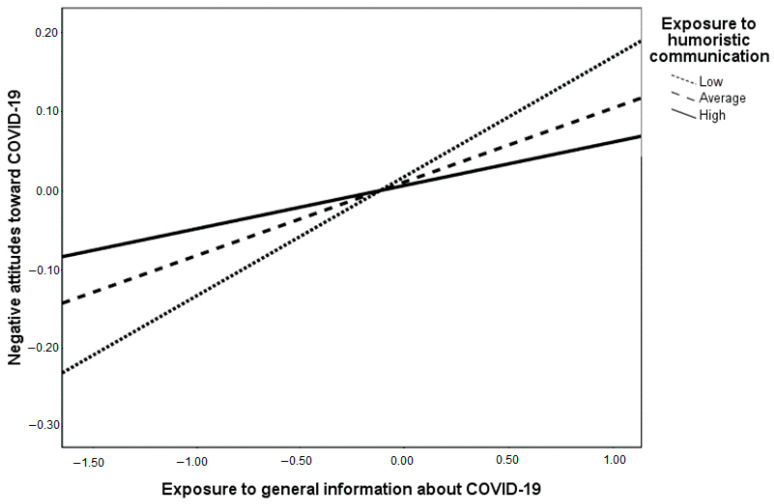
The interaction between exposure to general information and exposure to humoristic communication (EHC) on negative attitudes towards COVID-19. Note: conditional effects of exposure to general information about COVID-19 on negative attitudes are: B (unstandardized regression coefficient) = 0.15, SE (standard error) = 0.03, *p* < 0.001 for low EHC; B = 0.09, SE = 0.02, *p* < 0.001 for average EHC and B = 0.05, SE = 0.03, *p* = *0*.06 for high EHC.

Hypotheses 2, 3 and 4 focused on COVID-19 messaging as antecedents of negative attitudes and subjective norms. As indicated in Table 2, with respect to negative attitudes, the coefficient for exposure to general information about COVID-19 is positive and significant (β = 0.14, *p* < 0.001), therefore, Hypothesis 2a is supported. The coefficient for exposure to humoristic communication is negative, as predicted, yet it is not significant (β = −0.01, *p* = 0.81), therefore, Hypothesis 3a was not supported by the data. The coefficient for the interaction between exposure to general information and exposure to humoristic communication is negative and significant (β = −0.09, *p* = 0.01). The interaction effect is depicted in Figure 1 and it is aligned with Hypothesis 4a that predicted an attenuation effect of humoristic communication on the positive association between exposure to information about COVID-19 and negative attitudes towards COVID-19.

With respect to the subjective norms as a dependent variable, exposure to general information has a positive and marginally significant effect (β = 0.06, *p* = 0.08), therefore, Hypothesis 2b received limited support. Exposure to humoristic communication has a negative association with subjective norms, as predicted in Hypothesis 3b, yet it is not statistically significant (β = −0.03, *p* = 0.93). The interaction effect between exposure to general information and exposure to humoristic communication is negative yet not significant (β = −0.01, *p* = 0.73). We can therefore conclude that Hypothesis 4b was not supported.

**Figure 2:**
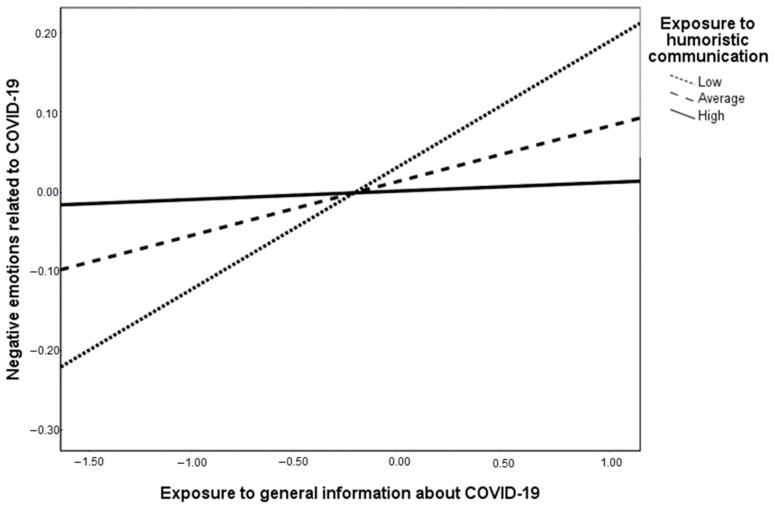
The interaction between exposure to general information and exposure to humoristic communication (EHC) on negative emotions in relation to COVID-19. Note: conditional effects of exposure to general information about COVID-19 on negative attitudes are: B = 0.15, SE = 0.05, *p* = 0.001 for low EHC; B = 0.07, SE = 0.04, *p* = 0.05 for average EHC and B = 0.01, SE = 0.04, *p* = 0.80 for high EHC.

With respect to negative emotions, the exposure to general information related to COVID-19 has a marginally significant effect (β = 0.07, *p* = 0.05), therefore, Hypothesis 2c received marginal support, while exposure to humoristic communication has a negative, yet not significant, effect (β = −0.02, *p* = 0.62), rejecting Hypothesis 3c. The interaction effect between exposure to general information and the exposure to humoristic communication is significant (β = −0.09, *p* = 0.01), and the conditional slopes depicted in Figure 2 show that, as predicted by Hypothesis 4c, the positive relation between exposure to general information about COVID-19 and negative emotions is attenuated by exposure to humoristic communication. We can therefore conclude that Hypothesis 4c was supported.

**Figure 3:**
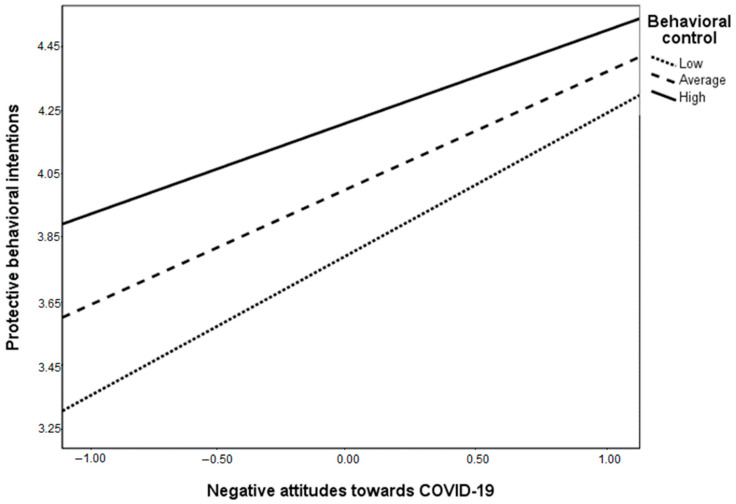
The interaction between negative attitudes towards COVID-19 and behavioral control in protective behavioral intentions. Note: BC = behavioral control; conditional effects of exposure to general information about COVID-19 on negative attitudes are: B = 0.51, SE = 0.05, *p* < 0.001 for low BC; B = 0.42, SE = 0.04, *p* < 0.001 for average BC and B = 0.33, SE = 0.05, *p* < 0.001 for high BC.

In Hypothesis 6, we expected a moderating effect of behavioral control on the relationship between negative attitudes, subjective norms and negative emotions on the one hand and the protective behavioral intentions on the other hand. The interaction effect between behavioral control and negative attitudes towards COVID-19 on protective behavioral intentions is negative and significant (β = −0.08; *p* = 0.02). The interaction effect is depicted in Figure 3 and, contrary to our expectations, behavioral control attenuates the positive association between negative attitudes towards COVID-19 and protective behavioral intentions. We can therefore conclude that, although the interaction effect between behavioral control and negative attitudes is significant, it is in the opposite direction than initially hypothesized, and, as such, Hypothesis 6a was not supported by the data. The interaction effect of subjective norms related to COVID-19 and behavioral control in protective behavioral intentions was not significant (β = 0.0001, *p* = 0.99), therefore, Hypothesis 6b was also not supported by the data. Finally, the interaction between negative emotions and behavioral control is also not significant (β = −0.04, *p* = 0.48), therefore, H6c is also not supported.

Hypothesis 5 focused on the mediating role of negative attitudes, negative emotions and subjective norms in relation to COVID-19 in the relationship between exposure to general COVID-19 messaging and the protective behavioral intentions. In order to capture both the mediating and the moderating effects hypothesized, we used the PROCESS macro (Model 21) [39] with negative attitudes, negative emotions and subjective norms as mediators, and exposure to humoristic communication as a moderator for the effect of exposure to general information on negative emotions, attitudes and subjective norms and with behavioral control as a moderator for the relationships between attitudes, subjective norms and negative emotions on the one hand and the preventive behavioral intentions on the other hand. The conditional indirect effects are summarized in Table 3 and Table 4. As indicated in Table 3, the indirect effect of exposure to general information on protective behavioral intentions, mediated by negative attitudes, is positive and significant only when exposure to humoristic information is low or average, therefore, we can conclude that Hypothesis 5a was supported. The conditional effects of exposure to general information, mediated by subjective norms, is not significant at any levels of the two moderators considered in the model, therefore, Hypothesis 5b received no support.

The conditional indirect effect of the exposure to general information on negative emotions is significant only when exposure to humoristic information is low, therefore, Hypothesis 5c was supported. Table 4 presents the overall indices of conditional moderated mediation, as well as the conditional indices of moderated mediation, taking into account the levels of behavioral control, and these indices are significant only for negative attitudes and negative emotions. Overall, the results show that only negative attitudes and negative emotions are significant mediators in the relationship between exposure to general information about COVID-19 and protective behavioral intentions. We can therefore conclude that Hypotheses 5a and 5c were supported, while Hypothesis 5b received no empirical support.

Because the data collected in our study were cross-sectional and no direct manipulation was used for our independent variable, we cannot refute the reversed causal chain, namely, that behavioral intentions impact on exposure to general information about COVID-19 via negative attitudes, negative emotions and subjective norms. We decided to test the original model without the moderators, as well as the reverse mediation model with behavioral intentions predicting attitudes, subjective norms and negative emotions, which in turn predict exposure to general information related to COVID-19. For the original model with no moderators, the total mediation effect when all mediators of the original model are included is significant (effect = 0.05, SE = 0.01, 95% CI = [0.02; 0.07]), the indirect effect via of negative attitudes is significant (effect = 0.04, SE = 0.01, 95% CI = [0.02; 0.06]), the mediation via subjective norms is not significant (effect = 0.003, SE = 0.003, 95% CI = [−0.0004; 0.01]) and the indirect effect via negative emotions is also significant (effect = 0.01, SE = 0.004, 95% CI = [0.0002; 0.02]). This pattern of results is fully aligned with the moderated mediation model reported earlier, supporting Hypotheses 5a and 5c. For the reverse mediation, we tested the extent to which negative attitudes, negative emotions and subjective norms mediate the relationship between behavioral intention and exposure to general COVID-19 information. The overall reversed mediation effect is not significant (effect = 0.03, SE = 0.03, 95% CI = [−0.04; 0.10]). Additionally, none of the separate mediation chains is significant and, for the mediation role of negative attitudes, the confidence interval includes zero (effect = 0.03, SE = 0.04, 95% CI = [−0.04; 0.11]), therefore, the indirect association is not significant. The same holds for subjective norms (effect = −0.002, SE = 0.02, 95% CI = [−0.03; 0.03]) and for negative emotions (effect = −0.002, SE = 0.02, 95% CI = [−0.04; 0.04]). This additional test shows that it is unlikely that the behavioral intentions influence exposure to COVID-19 general information via negative attitudes, subjective norms and negative emotions. Our hypotheses were framed starting from the theoretical sequencing specified in the TPB [9,17] and the models specifying the link between emotions and action [22,23,24], and the results of the reversed mediation tend to support such sequencing. However, the reversed mediation test is not a direct indication of the causal sequencing between the variables included in our study. An overview of the results in relation to the hypothesized relations is presented in Table 5.

## 4. Discussion

The aim of our study was to explore the interplay between exposure to general informative messages and exposure to humoristic messages in public health communication related to the COVID-19 pandemic. We have argued that exposure to general COVID-19 messaging, due to the threatening nature of the virus, generates negative evaluative tendencies, namely, negative attitudes, negative emotions and negative subjective norms, which in turn trigger protective behavioral intentions in relation to COVID-19. Our results support the assertions of the TPB as well as its usefulness in understanding protective behavioral intentions in the context of the COVID-19 pandemic [13,14,15,16] and show that attitudes, subjective norms and perceived behavioral control have positive effects on protective behavioral intentions. We also show that, next to the TPB predictors, negative emotions related to COVID-19 explain significant variance in protective behavioral intentions, a result that points to the relevance of taking into account emotional experiences in the general framework of the TPB. Our results further extend the insights of the TPB by showing that exposure to information concerning COVID-19 shapes the emergence of attitudes and subjective norms in relation to COVID-19. In particular, we show that exposure to general information related to COVID-19 generates negative attitudes towards the virus and the perception of a wide social consensus in relation to the virus, as well as the intention to engage in protective behaviors. These results are in line with previous reports showing that a better understanding of COVID-19 is an important predictor for attitudes and social norms [16]. Moreover, our results point towards the detrimental effect of humoristic communication in the context of the COVID-19 pandemic. Exposure to such humoristic messages attenuates the positive association between exposure to general information and negative emotions, as well as attitudes towards COVID-19. This result offers a plausible explanation for the negative association between the use of social media and intention to engage in protective behaviors [5,7], as, most likely, the exposure to humoristic information occurs via social media rather than official media channels. The exposure to humoristic messages does not significantly decrease negativity (attitudes or emotions) towards COVID-19, as expected. The sense of urgency created by national measures implemented at the time of the study could have prevented such a negative effect. The moderating role of humor is, however, relevant for communication reasons in that it seems that humoristic messages dilute the expected effect of exposure to generic COVID-19 information on public negative attitudes towards COVID-19.

In addition, we show that behavioral control moderates the association between negative attitudes towards COVID-19 and protective behavioral intentions. This moderation effect is, however, not in line with the hypothesis derived from the TPB, such that behavioral control attenuates rather than accentuates the association between the negative attitudes and protective behavioral intentions. This interaction effect could be explained by the individual differences in which perceptions of behavioral control are rooted. The self-confidence could, for example, explain why, for strong beliefs of behavioral control, the association between attitudes and protective behavioral intentions is weaker. Behavioral control, however, has a positive and significant effect on intention to engage in protective behaviors. It is just that the interaction with the negative attitudes toward COVID-19 yields surprising effects. Future research could continue to extend the TPB [16] and explore whether such individual differences or perception biases could explain such an association.

Our results show that Romanian-speaking participants report more negativity (negative attitudes, emotions and subjective norms) in relation to COVID-19 than Kazakh participants. This effect is certainly not due to different exposure to information, as this effect is entered in the regression analysis as well. A plausible explanation is that the epidemiological impact at the time of the study was greater in Romania than in Kazakhstan, as at the time of closing the study, Romania had 762 cases with 17 deaths while Kazakhstan had only 435 officially reported cases with only three deaths. As the number of cases and the number of deaths were frequently reported in mass media, it is likely that the higher negativity towards COVID-19 in Romania was due to the magnitude of the epidemiological impact, as reflected in media reports. Romanian respondents however, report lower scores on protective behavioral intentions, therefore it is likely that other factors (such as cultural norms, habits, trust in media) explain the engagement in protective behavioral intentions.

### 4.1. Limitations

Our study is based on self-reports and, as such, the results are susceptible to common method bias. Simulation studies, however, show that common method bias is less likely to lead to an overestimation of interaction effects [40,41]. As the overall model tested in our paper is a moderated mediation model, we can conclude that although the mediation effects could be overestimated due to the fact that variables were evaluated using the same source, the interaction effects are less likely to be biased. Meta-analytical evidence suggests that when the relationships stipulated in the TPB are based on self-reports only, the percentage of variance explained in behavioral intentions is higher than if data are collected from different sources [10,12]. A second limitation is the convenience sampling used in the study. The time constraints did not allow for a better approach to sampling, and given the convenient sample used in our study (with an unbalanced gender distribution) we cannot generalize our findings. The results are, however, associated with the trends reported in various meta-analytical studies investigating the usability of the TPB [10,11,12,19]. Therefore, we do not expect that the relationships between the TPB variables will be much different in other samples that investigate COVID-19-related issues. Although the predictions of the TPB are expected to hold in other contexts and samples, it is important to explore potential differences in how attitudes and social norms differ, for example, in healthcare professionals that have direct contact with COVID-19 patients. Third, for our exposure to information measure, we have used a single-item and the use of single items raises concerns about the validity of the findings. When the content being measured is clear and non-ambiguous [42,43,44,45], as it was in the case of exposure to information in the last 14 days, we believe that the implications of using single-item measures for the validity of our findings are not that serious. Fourth, protective behavioral intentions were the main dependent variable in our study, therefore, no clear inference can be made concerning the engagement in real protective behaviors against COVID-19. Based on meta-analytic evidence showing a clear causal association between change in behavioral intentions and subsequent real behavioral change [46], we can be confident that protective behavioral intentions against COVID-19 are positively correlated with real engagement in such behaviors, yet, based on our data, this inference cannot be fully supported. Fifth, in our study, in order to illustrate the difference between exposure to general and humoristic information related to COVID-19, we used two examples extracted from different media channels, namely, the official governmental sites for the general COVID-19 information and social media for the humoristic information. We did not explicitly ask participants to evaluate the extent to which they are exposed to social media or official media messaging, yet we asked them to evaluate the extent to which they were exposed to similar messages in the past 14 days. Although the aim of our study was not to explore the differential exposure to information stemming from different media channels in the context of the COVID-19 pandemic, the use of the two examples extracted from different media channels could have conflated with the distinction between general and humoristic information. Although such a conflation is a possible source of error in our study, it is likely to actually have caused a congruent conflation situation, as very rarely, if ever, do official channels communicate humoristic information related to COVID-19. Future studies could, for example, try to disentangle the extent to which participants are exposed to general versus humoristic information stemming from social media alone. We did not explicitly investigate the difference between different social media channels used by participants and such an investigation could have yielded more insights into the role of media exposure and attitudes towards COVID-19. Additionally, because social media is more likely to perpetuate misinformation than official communication channels [5,7,8], future studies could disentangle the effect of humor and misinformation in social media communication. Sixth, we formulated hypotheses in line with the TPB, yet due to the cross-sectional nature of our study, we cannot draw definite conclusions about the causal sequencing in our serial model. By testing a reverse causation model, we show that the association between behavioral intentions and exposure to general COVID-19 messaging is not likely to be explained by negative emotions, attitudes and subjective norms. Definite causal claims could be made only under a clear participant randomization in experimental conditions that manipulate the negative attitudes, emotions and subjective norms in relation to COVID-19. Future research could also explore other variables, such as income, social status and political beliefs that could also impact on compliance with the protective behaviors during pandemics. Finally, our results show that although the scores for protective behavioral intentions range from 1 to 5 (therefore, we have no range restriction on this variable), the average score is rather high for this variable, therefore, a ceiling effect could have influenced our results.

### 4.2. Practical Implications

Our study has important practical insights for public communication in the context of the COVID-19 pandemic. We will first focus on the emergent findings concerning the non-hypothesized associations. According to our results, age is positively associated with the negative attitudes towards COVID-19, yet is has no significant association with protective behavioral intentions. The study was carried out in national contexts in which governments issued clear regulations concerning adults older than 65 years (especially in Romania), and our results show that more (communicative and persuasive) attention should be devoted to this population segment. Overall, the exposure to general information about COVID-19 has a direct positive effect on the likelihood of engaging in protective behaviors, therefore, more attention should be paid to the vulnerable age groups. Social media could also help the timely dissemination of accurate information concerning the preventive and curative measures to combat the pandemic [47], especially if special attention is given to reaching out to older adults that may lack digital skills to search for and accurately analyze information received from various channels. Another result concerned the higher propensity of women towards protective behavioral intentions, and, although this result is aligned with previous research on gender differences in risk taking and agency, we believe that better communicative and persuasive messages could also be directed towards men. According to our results, education is a beneficial factor for the intention to engage in protective behaviors. Various explanations are possible concerning this association, yet a clear practical message is that the communicative and persuasive messages should focus on population segments that are less educated. Such individuals may also score lower in digital literacy and, as such, have difficulty in being up to date with the information on the COVID-19 pandemic, the necessary protective measures and behaviors. Future research should find ways in which people with lower digital literacy can be helped to discern the validity of information they receive from various media channels and to select trustworthy sources to inform their decisions and actions.

A particularly relevant outcome of our study concerns the positive association between the survey completion date and negative attitudes and subjective norms. During the days in which the study was conducted, regulations in both countries were strict, based on a state of national emergency and social distancing, and limitations on personal mobility were reinforced in both countries as the study proceeded. Our results show that such measures were most certainly effective, as far as attitude change was concerned, because respondents that filled in the survey in the first days reported significantly less negative attitudes and subjective norms than the ones that filled in the survey towards the end of the study. However, the association between the survey completion date and intentions to engage in protective behaviors is not significant. Apparently, such restrictive measures increased negativity towards the COVID-19 pandemic, yet they did not seem to strengthen the intention to engage in protective behaviors. Another plausible explanation for these results is the epidemiological impact, widely reported in the media that increased in time as the data collection progressed and it was larger in Romania than in Kazakhstan, offering a plausible interpretation for the negativity towards the pandemic. More in-depth analyses are needed, on how the epidemiological impact is presented to the public and how the restrictive measures are communicated and implemented, as they steer negativity, and may not have the desired effect on behavioral intentions.

Finally, our results have implications concerning the use of humor in public health communication. Although, in general, the use of humor is praised in a variety of settings, our results show that exposure to humor may decrease the association between the exposure to general information and the negativity towards COVID-19. The use of humor in public health communication should be cautioned, as these effects indirectly impact the likelihood to engage in protective behaviors. It could be that joking helps in general, yet, in this pandemic, exposure to humoristic communication may indirectly dilute the intended effect of official communication on protective behavioral intentions.

### 4.3. Ethics Statement

The study was approved by the Ethics Review Board of Babes-Bolyai University, Cluj-Napoca, Romania. Participants were invited via social media sites to take part in the study using a snowball approach and they were informed about the aim of the study. Participation was anonymous, participants could withdraw from the study at any moment and they were assured that data will remain confidential and unidentified.

## 5. Conclusions

Our study builds on the Theory of Planned Behavior [9] to explore antecedents of protective behavioral intentions at the onset of the COVID-19 pandemic in 2020. We collected data in a sample of Romanians and Kazakhs and our results show that negativity in relation to the COVID-19 pandemic, reflected by negative attitudes, negative perceived social norms and negative emotions, positively predicts the protective behavioral intentions. Behavioral control has a positive effect on protective behavioral intentions and contrary to what was hypothesized, it attenuates rather than accentuates the positive association between negative attitudes and the protective behavioral intentions. The negative attitudes and emotions in relation to COVID-19 are positively predicted by exposure to general information about COVID-19 and this relation is attenuated by exposure to humoristic communication in relation to the pandemic. The association between exposure to general information and protective behavioral intentions is mediated by negative attitudes and emotions, especially when exposure to humoristic communication is low. Although survey completion day correlates positively with negativity towards COVID-19, it does not predict the protective behavioral intentions. It is likely that the magnitude of the epidemiological impact reported in the media augments the negativity towards the pandemic, yet it does not necessarily impact on protective behavioral intentions.

## Figures and Tables

**Table 1:** Means, standard deviations and correlations.

Variables	Mean RO	SD RO	1	2	3	4	5	6	7	8	9	10	11	Mean KZ	SD KZ
1. Age	35.06	10.42	1	−0.06	0.37 **	0.07	−0.22 **	−0.01	0.13	−0.06	0.00	0.13	0.11	33.91	13.72
2. Gender	0.78	0.43	0.02	1	0.09	−0.40 **	0.18 *	0.05	−0.13	−0.17 *	0.38 **	−0.09	0.12	0.63	0.48
3. Education	4.43	1.32	0.37 **	0.01	1	−0.15 *	0.06	0.08	0.03	−0.04	0.08	−0.05	0.11	4.03	1.30
4. Survey completion day	3.12	2.16	−0.09 *	−0.13 **	−0.10 *	1	−0.21 **	0.06	0.18 *	0.15 *	−0.23 **	0.18 *	0.07	9.50	4.27
5.Exposure to humoristic information related to COVID−19	6.07	1.51	−0.07	−0.04	−0.12 **	0.04	1	0.25 **	0.01	−0.10	0.11	0.06	0.06	5.75	1.48
6. Exposure to general information related to COVID−19	6.24	1.19	0.04	0.06	0.08	0.05	0.08	1	0.10	0.00	0.12	−0.01	0.09	6.23	1.01
7. Negative attitudes towards COVID−19	3.69	0.76	0.10 *	0.03	0.13 **	0.16 **	−0.02	0.18 **	1	0.28 **	−0.02	0.43 **	0.39 **	3.28	0.82
8. Subjective norms related to COVID−19	3.82	0.71	0.09 *	−0.01	0.13 **	0.15 **	−0.02	0.10 *	0.47 **	1	−0.04	0.15 *	0.11	3.61	0.80
9. Behavioral control	3.54	0.91	0.01	0.03	−0.07	0.08	0.04	0.02	0.03	0.09 *	1	−0.24 **	0.38 **	3.32	0.99
10. Negative emotions related to COVID−19	4.81	1.06	0.07	0.05	0.01	0.14 **	−0.06	0.11 **	0.34 **	0.18 **	−0.03	1	0.26 **	4.29	1.28
11. Protective behavioral intentions	4.01	0.77	0.01	0.12 **	0.11 **	0.09 *	−0.02	0.29 **	0.52 **	0.33 **	0.22 **	0.31 **	1	3.94	0.81

Note. Correlation coefficients are presented in the table with the scores for the Kazakhstan sample above the diagonal and means and standard deviations presented in the last column; gender was coded as a dummy variable, with 1 = women and 0 = men; the values 1–11 represent variables; SD: standard deviation; RO = Romania and KZ = Kazakhstan; * *p* < 0.05; ** *p* < 0.01; for demographic variables, significant country-level differences were observed for gender (a significantly higher percentage of men respondents in the KZ sample) and education (F_(1, 773)_ = 22.67, *p* < 0.001, η_p_^2^ = 0.02, observed power = 0.95, with respondents in RO reporting higher education levels than the participants in KZ), while no significant age differences were observed across the respondents from the two countries (F_(1, 773)_ = 1.27, *p* = 0.26, η_p_^2^ = 0.002, observed power = 0.20).

**Table 2:** Results of the regression analyses with robust standard errors.

Variable	Negative Attitudes		Subjective Norms		Negative Emotions		Protective Behavioral Intentions	
	Model 1	Model 2	Model 1	Model 2	Model 1	Model 2	Model 1	Model 2
Constant	2.45 ***(0.17)	2.44 *** (0.17)	3.09 ***(0.16)	3.10 *** (0.15)	3.38 *** (0.25)	3.37 *** (0.25)	3.77 *** (0.15)	3.74 *** (0.15)
Age	0.01 * (0.003)	0.01 * (0.003)	0.01 (0.003)	0.01 (0.003)	0.01 ** (0.004)	0.01 ** (0.004)	−0.004 (0.002)	−0.004 (0.002)
Gender	0.03 (0.07)	0.03 (0.07)	−0.05 (0.06)	−0.05 (0.06)	0.11 (0.10)	0.11 (0.10)	0.16 ** (0.06)	0.16 ** (0.06)
Education	0.05 * (0.02)	0.05 * (0.02)	0.05 * (0.02)	0.05 * (0.02)	−0.03 (0.03)	−0.03 (0.03)	0.05 * (0.02)	0.05 * (0.02)
Country	0.67 ***(0.09)	0.67 *** (0.09)	0.43 *** (0.09)	0.43 *** (0.09)	0.92 *** (0.13)	0.92 *** (0.13)	−0.20 * (0.08)	−0.18 * (0.08)
Survey completion day	0.05 ***(0.01)	0.05 *** (0.01)	0.04 *** (0.01)	0.04 *** (0.01)	0.06 *** (0.02)	0.06 *** (0.02)	0.01 (0.01)	0.01 (0.01)
Exposure to general information (EGI)	0.10 ***(0.03)	0.09 *** (0.02)	0.04 ^†^ (0.02)	0.04 ^†^ (0.02)	0.07 * (0.04)	0.07 * (0.04)	0.10 *** (0.02)	0.10 *** (0.02)
Exposure to humoristic communication (EHC)	−0.002 (0.02)	−0.0041 (0.02)	−0.02 (0.02)	−0.02 (0.02)	−0.01 (0.03)	−0.01 (0.03)	−0.01 (0.02)	−0.01 (0.02)
EGIxEHC		−0.04 * (0.02)		−0.005 (0.02)		−0.06 * (0.02)		
Negative attitudes (NA)							0.37 *** (0.04)	0.37 *** (0.04)
Subjective norms (SN)							0.06 ^†^ (0.04)	0.06 ^†^ (0.04)
Behavioral control (BC)							0.21 *** (0.03)	0.25 *** (0.04)
Negative emotions (NE)							0.10 *** (0.02)	0.11 *** (0.02)
NAxBC								−0.08 * (0.04)
SNxBC								0.0001 (0.03)
NExBC								−0.02 (0.03)
N	735	735	735	735	735	735	735	735
R²	0.11	0.12	0.05	0.05	0.08	0.09	0.37	0.38
F change	12.98 ***	6.10 *	5.25 ***	0.12	8.92 ***	6.46 *	37.97 ***	3.77 *

Note. Unstandardized regression coefficients are presented in the table; gender was coded as a dummy variable, with 1 = women and 0 = men; country was coded as a dummy variable, Romania = 1 and Kazakhstan = 0; Model 1 reports the main effects and Model 2 includes interaction effects; N=sample size for the analyses; R² =the R squared value; ^†^ *p* < 0.10; * *p* < 0.05; ** *p* < 0.01; *** *p* < 0.001.

**Table 3:** Conditional indirect effects for the two moderators.

Moderators and Levels		Negative Attitudes		Subjective Norms		Negative Emotions	
EHI	BC	Effect (SE)	95% CI	Effect (SE)	95% CI	Effect (SE)	95% CI
Low	Low	**0.07 (0.02)**	**[0.04; 0.11]**	0.003 (0.004)	[−0.003; 0.01]	**0.02 (0.01)**	**[0.003; 0.04]**
Low	Average	**0.06 (0.01)**	**[0.03; 0.09]**	0.003 (0.003)	[−0.001; 0.01]	**0.02 (0.02)**	**[0.003; 0.03]**
Low	High	**0.04 (0.01)**	**[0.02; 0.07]**	0.003 (0.004)	[−0.002; 0.01]	**0.01 (0.01)**	**[0.002; 0.03]**
Average	Low	**0.04 (0.01)**	**[0.02; 0.07]**	0.003 (0.003)	[−0.001; 0.01]	0.01 (0.01)	[−0.0005; 0.02]
Average	Average	**0.03 (0.01)**	**[0.02; 0.06]**	0.003 (0.003)	[−0.002; 0.01]	0.01 (0.004)	[−0.0005; 0.02]
Average	High	**0.03 (0.01)**	**[0.01; 0.05]**	0.003 (0.003)	[−0.002; 0.01]	0.01 (0.01)	[−0.0004; 0.02]
High	Low	0.02 (0.02)	[−0.004; 0.06]	0.002 (0.003)	[−0.003; 0.01]	0.001 (0.01)	[−0.001; 0.01]
High	Average	0.02 (0.01)	[−0.003; 0.04]	0.002 (0.003)	[−0.002; 0.01]	0.001 (0.004)	[−0.001; 0.01]
High	High	0.02 (0.01)	[−0.002; 0.04]	0.002 (0.003)	[−0.003; 0.01]	0.0001 (0.004)	[−0.001; 0.01]

Note: EHI—exposure to humoristic information; BC—behavioral control; SE –standard error; CI—confidence interval; significant effects are presented in bold (the effects for which the 95% CI does not include zero).

**Table 4:** Indices of conditional moderated mediation for levels of behavioral control.

Behavioral Control	Negative Attitudes		Subjective Norms		Negative Emotions	
	Effect (SE)	95% CI	Effect (SE)	95% CI	Effect (SE)	95% CI
Low	**−0.02 (0.01)**	**[−0.03; −0.003]**	−0.003 (0.001)	[−0.004; 0.002]	**−0.01 (0.004)**	**[−0.02; −0.0001]**
Average	**−0.01 (0.01)**	**[−0.03; −0.002]**	−0.003 (0.001)	[−0.003; 0.002]	**−0.01 (0.004)**	**[−0.01; −0.0001]**
High	**−0.01 (0.01)**	**[−0.02; −0.002]**	−0.003 (0.001)	[−0.004; 0.002]	−0.01 (0.003)	[−0.01; 0]

Note: CI—confidence interval; significant effects are presented in bold (the effects for which the 95% CI does not include zero).

**Table 5:** An overview of the hypotheses tested in the study.

Hypotheses	Status
H1: Negative attitudes (H1a), subjective norms (H1b), behavioral control (H1c) and negative emotions (H1d) have a positive association with behavioral intentions to prevent infection with COVID-19.	H1a, H1c and H1d—supported H1b—marginally supported
H2: Exposure to general information concerning COVID-19 is positively associated with negative attitudes (H2a), subjective norms (H2b) and negative emotions (H2c) related to COVID-19.	H2a—supported H2b and H2c—marginally supported
H3: Exposure to humoristic information about COVID-19 is negatively associated with negative attitudes (H3a), subjective norms (H3b) and negative emotions (H3c) related to COVID-19.	H3a, H3b and H3c—not supported
H4: Exposure to humoristic information about COVID-19 attenuates the positive effect of exposure to general information on negative attitudes (H4a), subjective norms (H4b) and negative emotions (H4c) in relation to COVID-19.	H4a and H4c—supported H4b—not supported
H5a: Negative attitudes towards COVID-19 mediate the effect of exposure to general information on behavioral intentions to prevent infection with COVID-19.	H5a—supported
H5b: Subjective norms towards COVID-19 mediate the effect of exposure to general information on behavioral intentions to prevent infection with COVID-19.	H5b—not supported
H5c: Negative emotions experienced in relation to COVID-19 mediate the effect of exposure to general information on behavioral intentions to prevent infection with COVID-19.	H5c—supported
H6a: Behavioral control accentuates the positive association between negative attitudes towards COVID-19 and protective behavioral intentions.	H6a—not supported
H6b: Behavioral control accentuates the positive association between the subjective norms and protective behavioral intentions.	H6b—not supported
H6c: Behavioral control accentuates the positive association between negative emotions experienced in relation to COVID-19 and protective behavioral intentions.	H6c—not supported

## Data Availability

Data is available upon substantiated request from the corresponding author.
